# Acute effects of combined cycling and plyometrics on vertical jump performance in active males

**DOI:** 10.5114/biolsport.2023.119989

**Published:** 2022-11-16

**Authors:** Fernando González-Mohíno, Victor Rodrigo-Carranza, Sergio Rodríguez-Barbero, Anthony Turner, José María González-Ravé

**Affiliations:** 1 Sports Training Laboratory, Faculty of Sports Sciences, Universidad de Castilla-La Mancha, Spain; 2 Facultad de Ciencias de la Vida y de la Naturaleza, Universidad Nebrija, Madrid, España; 3 Sport, Physical Education & Health Sciences, University of Edinburgh, UK

**Keywords:** Myosin light chain, Warm-up, Power

## Abstract

The aim of this study was to analyze the acute effects of high vs low-intensity cycling efforts, combined with plyometrics, on vertical jump performance. Twenty-four physically active men (mean ± SD: 23 ± 2 years, 72.1 ± 10.1 kg, 1.73 ± 0.07 m) were randomly divided into two groups: experimental group (EXP, n = 16) and control group (CON, n = 8). EXP competed 2 experimental trials in a random order: (a) short high-intensity interval exercise (HI + Plyo) [5 × 10 s of cycling (“all-out”)/50 s active rest] or (b) low-intensity continuous exercise (LO + Plyo) [5 min of cycling at 75% of the HR_max_)], along with 3 × 10 plyometric bounds (drop jumps)/1 min rest between sets. CON used a preconditioning activity of 13 min of low intensity cycling at ~60% of HR_max_. Both EXP interventions significantly increased (p ≤ 0.05) the countermovement jump (CMJ) height at 1 min, 3 min, 6 min and 9 min compared to baseline, while the CON remained unchanged. There were no significant differences in CMJ performance enhancement between HI + Plyo (largest 11.2% at 9 min) and LO + Plyo (largest 15.0% at 3 min) at any time-point, suggesting that the plyometric component may be most important, with HR recovery taking slightly longer following HI + Plyo. The findings suggest that CMJ performance can be enhanced following high or low-intensity cycling combined with plyometric preconditioning activities in active males, the optimum recovery period likely to be individual-specific.

## INTRODUCTION

The ability to develop muscular power (energy output per unit of time) is critical to successful outcomes in many sports events, such as sprint races, long and high jumps and other sporting actions like swimming starts or accelerations during team-based sports [1]. Some activities or actions (e.g., sprint, heavy-load exercises or stretching) performed before sport (warm-up) have shown acute improvements in subsequent performance [2, 3]. A proposed mechanism under-pinning such acute performance enhancement is post-activation potentiation (PAP). Several authors have demonstrated that muscle PAP is a phenomenon that can acutely increase muscular power and, consequently, performance [4, 5]. PAP might cause gains in power after heavy muscle preloading as a result of myosin light chain phosphorylation and increased recruitment of higher order motor units [6]. However, the efficacy of PAP mechanisms to enhance performance ultimately depends on the balance between competing and simultaneous fatigue and PAP phenomena [6]. This balance is affected by many factors, including training experience of the athletes [4, 7], recovery period [8] and the intensity of the conditioning activity performed [9].

Regarding the intensity of the conditioning activity, most studies have reported positive effects when lifting heavy weights [10, 11] or plyometric exercises prior to explosive movements (sprints or vertical jumps) [8, 12, 13]. However, while strength and plyometric exercises are generally utilized as a potentiating stimulus, less is known about the combination of these exercise modes with endurance exercise that is often also included in warm-ups (e.g., low or high-intensity cycling efforts). In addition, the post-activation performance enhancement (PAPE) concept has more recently been proposed [14, 15] for use after a voluntary muscular performance enhancement related to a high-intensity contractions, without confirmatory evidence of classical PAP, that is often not measured. Both phenomena can occur at different time points [16, 17] and PAPE was not observed despite PAP being evoked [18, 19]. For this reason, it is important to consider the time point at which an improvement of performance occurs after a conditioning activity. A recent meta-analysis [20] found that sufficient recovery occurs after 3–7 min in trained athletes. However, recreational athletes or with lower experience may need more time to recover from high-intensity protocols [16, 21]. For that reason, it is crucial to investigate the optimal recovery time for these athletes according to the preconditioning activity.

Therefore, the main aim of this study was to analyze the acute effects of high vs low-intensity cycling efforts, combined with plyometrics, on markers of vertical jump performance and heart rate. The second aim was to analyze the influence of recovery time post-stimulus. We hypothesized that the combination of cycling with plyometrics would enhance jump performance compared to a control condition, but with a differing time-course of PAPE effects for high-intensity vs. low-intensity cycling conditions.

## MATERIALS AND METHODS

### Subjects

Twenty-four physically active men (mean ± SD: 23 ± 2 years, 72.1 ± 10.1 kg, 1.73 ± 0.07 m) participated in this study. Subjects were non-smokers, free from any pre-existing medical conditions and musculoskeletal injuries. They performed varied sporting activities (“gym”-based training, endurance exercises such as running and swimming, and various sports such as soccer and basketball), habitually exercising for > 6 h per week.

Using an online randomization tool, subjects were randomly divided into two groups: experimental group (EXP, n = 16; mean ± SD: 23 ± 2 years; 72.8 ± 10.9 kg and 1.73 ± 0.08 cm) and control group (CON, n = 8; mean ± SD: 23 ± 2 years; 70.6 ± 8.8 kg and 1.74 ± 0.06 cm). Prior to the study, all participants were informed about the testing protocols, possible risks involved and were invited to provide written informed consent. The study was performed in accordance with the principles of the Declaration of Helsinki (October 2008, Seoul), and the experimental protocols were approved by the local ethics committee.

### Experimental design

A randomized and crossover study design was used to compare the effects of cycling efforts (high vs low-intensity) along with plyometrics on vertical jump performance and heart rate (HR) compared to a control condition. EXP competed 2 experimental trials, (a) HI + PLYO – short high-intensity interval exercise [5 × 10 s of cycling (“allout”)/50 s active rest] or (b) LO + PLYO – low-intensity continuous exercise [5 minutes of cycling at 75% (range 73–82%) of the HR_max_, 220-age)], along with 3 sets of 10 plyometric (PLYO) bounds (drop jump [DJ]) with 1 min rest between sets, timings previously used by Turner et al. [13] with horizontal bounds. The subjects of the HI + PLYO condition were strongly encouraged to maintain a maximal effort (all-out) throughout the cycling exercise. Both experimental conditions were separated by 5 days. On the first testing day, CON performed a preconditioning activity which consisted of 13 minutes (time matched to experimental conditions) of low-intensity cycling at 60% (range: 59.4–63.5%) of HR_max_ as an example of general warm-up [22].

### Procedure

All subjects visited the laboratory on two different days with 5 days apart and without performing heavy exercise in the 48 h prior to the visits. During the first visit height was measured to the nearest 0.1 cm with a portable stadiometer and body mass was measured using a calibrated balance beam scale (Seca, Bonn, Germany) to the nearest 0.1 kg. In addition, subjects completed a short survey about their previous experience in strength and endurance training (hours and days of training per week, years of experience, etc.). To control the possible PAPE effect, the following considerations were taken [23]: 1) all subjects were familiarized with the countermovement jump (CMJ) and before each warm-up condition, three maximal CMJ were performed; 2) the warm-up conditions were randomized; 3) time of day, diet, hydration, and physical activity performed and use of caffeine were controlled in the days prior to testing. Subjects refrained from caffeine use before each training session.

**FIG. 1:**
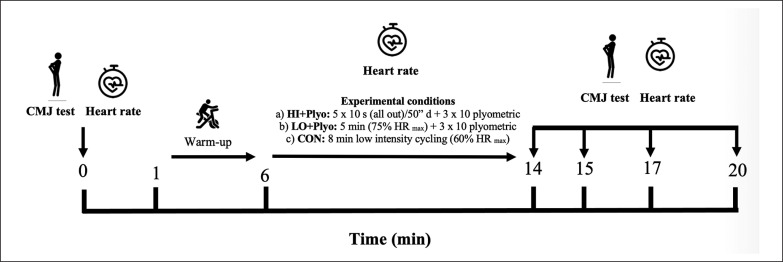
Overview of experimental design, indicating the protocols and timing of measurements.

Then, a CMJ was performed on a force platform Kistler Quattro-Jump (Kistler, Switzerland) at 500 Hz considered as the *gold standard* that served as baseline [24]. The force platform was calibrated according to the manufacturer’s recommendations. The order of EXP conditions for each subject was determined by block randomization using an online randomization tool. After baseline CMJ, all groups performed the preconditioning activities after a standardized 5 min of cycling at 60% HR_max_. Finally, CMJ and HR were measured at 1 min, 3 min, 6 min and 9 min of rest (Figure 1) to profile both transient fatigue and potentiation effects. Before the CMJ assessment, the subjects stood motionless on the force platform to measure their body weight [25]. Participants were instructed to perform each vertical jump “as high as possible”, with both hands placed on the hip followed by a rapid decent to a self-determined squat depth. CMJ performance variables assessed were jump height (cm), relative maximal force (%BW), average power (Watts), average force (N) and average velocity (m/s) of the CMJ were recorded from a force plate. The selected variables have been described as used to assess explosive leg muscle function [26]. These variables were automatically calculated through take-off velocity using MARS software (Kistler, Winterthur, Switzerland).

The HI and LO cycling were performed on the same cycle ergometer (Technogym, Gambettola, Italy) and the plyometrics jumps from the same box of 40 cm height (Technogym, Gambettola, Italy). Finally, HR was measured continuously during the preconditioning activities and recovery periods using a Polar^®^ watch (Vantage M, Polar, Kempele, Finland) and a heart rate sensor with a chest band (H10 sensor, Polar, Kempele, Finland). The overview of experimental design is shown in Figure 1.

### Statistical analysis

Statistical analyses were performed using SPSS software (version 21; SPSS, Inc, Chicago, IL, USA). Data are presented as mean ± SD. Significance was set at p ≤ 0.05. Data were screened for normality of distribution and homogeneity of variance using a Shapiro-Wilk Normality Test. Two-way (3 × 5) mixed analyses of variance (between-groups factors: condition [HI + Plyo, LO + Plyo, CON] × time [within-groups factors: baseline, 1 min, 3 min, 6 min and 9 min]) were used. Mauchly’s test was consulted and if sphericity was violated, Greenhouse-Geisser correction was applied. Post-hoc Bonferroni adjustment was applied for post-hoc pairwise comparisons. We calculated the effect size using the partial eta squared (ŋ^2^). Values of 0.01, 0.06 and above 0.15 were considered as small, medium, and large, respectively [27].

## RESULTS

The results for the CMJ parameters are displayed at the Table 1.

**TABLE 1:** Results for countermovement jump performance during the study for each warm-up condition.

	Time				

	Baseline	1 min post	3 min post	6 min post	9 min post
CMJ HI + Plyo (cm)	33.46±5.99	35.84±6.03*	35.88±5.81*	36.87±6.57**	37.21±6.38***
CMJ LO + Plyo (cm)	34.14±6.68	38.14±6.68***	39.29±6.94***	39.25±7.63***	38.84±7.52**
CMJ CON (cm)	36.39±7.65	37.50±7.81	37.54±7.64	38.31±7.27	38.57±7.15
RMF HI + Plyo (%BW)	228.63±24.07	242.11±30.60	242.02±29.34	241.02±30.66	244.90±30.10
RMF LO + Plyo (%BW)	231.71±25.68	243.90±29.71	239.39±30.09	241.07±25.84	242.33±28.43
RMF CON (%BW)	232.81±21.80	240.86±22.03	243.10±22.79	243.00±26.34	246.98±24.86
Power HI + Plyo (W)	1847.60±414.51	1903.69±515.29	1926.07±455.47	1943.33±446.84	1955.47±483.44
Power LO + Plyo (W)	1948±430.32	2102.32±461.82	2107.43±485.98	2084.64±457.18	2053.10±468.14
Power CON (W)	2011.77±351.22	2023.19±442.98	2098.77±348.08	2071.85±332.52	2109.31±365.40
Force HI + Plyo (N)	1296.61±249.56	1331.46±223.10	1332.78±249.78	1265.27±406.27	1356.30±263.41
Force LO + Plyo (N)	1344.65±252.04	1381.13±256.52	1387.30±268.45	1376.37±243.51	1362.25±256.78
Force CON (N)	1367.62±155.98	1396.00±155.58	1415.31±168.15	1387.32±151.80	1407.54±156.45
Velocity HI + Plyo (m/s)	1.51±0.16	1.54±0.18	1.56±0.15	1.58±0.15	1.57±0.19
Velocity LO + Plyo (m/s)	1.54±0.17	1.61±0.18	1.61±0.18	1.61±0.18	1.60±0.19
Velocity CON (m/s)	1.57±0.14	1.60±0.14	1.60±0.16	1.59±0.17	1.63±0.18

Data are presented as mean ± standard deviation.					

CMJ countermovement jump, HI short high intensity interval exercise, LO low intensity continuous exercise, CON control condition, Plyo Plyometrics, RMF relative maximal force, %BW % body weight, W watts, bracket = all conditions different from baseline,

* p < 0.05;

** p < 0.01;

*** p < 0.001.

**FIG. 2:**
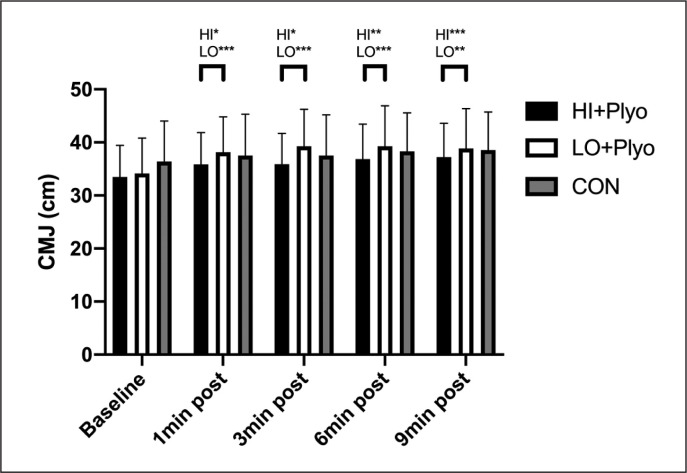
Results for CMJ for each warm-up condition. $ = Different from control condition, bracket = all conditions different from baseline. *p ≤ 0.05; **p ≤ 0.01; ***p ≤ 0.001.

There was no main effect of condition on CMJ height (F = 2.668, p = 0.548, partial ŋ^2^ = 0.029). However, there was a significant main effect of time (F = 39.103, p ≤ 0.001, partial ŋ^2^ = 0.488) and a significant time × condition interaction effect (F = 17.536, p ≤ 0.05, partial ŋ^2^ = 0.155), with large effect sizes. As shown in Table 1 and Figure 2, post-hoc comparisons revealed that CMJs were significantly enhanced (p ≤ 0.05) at 1 min, 3 min, 6 min and 9 min compared to baseline in HI + PLYO and LO + PLYO, with no significant differences in CON. There were no significant differences between EXP conditions at any time of recovery.

There was no main effect of condition on relative maximal force (F = 0.017, p = 0.983, partial ŋ^2^ = 0.001), with no time × condition interaction effect (F = 0.377, p = 0.929, partial ŋ^2^ = 0.038). However, there was a main effect of time (F = 6.365, p ≤ 0.001, partial ŋ^2^ = 0.408), with large effect size. Relative maximal force was significantly higher at 1 min, 3 min, 6 min and 9 min compared to baseline (p ≤ 0.01).

Similarly, there was no main effect of condition on average power (F = 0.661, p = 0.522, partial ŋ^2^ = 0.033), nor a time × condition effect (F = 0.913, p = 0.508, partial ŋ^2^ = 0.045). There was a main effect of time (F = 9.585, p ≤ 0.001, partial ŋ^2^ = 0.197), with large effect size. Average power was significantly higher at 1 min, 3 min, 6 min and 9 min compared to baseline (p ≤ 0.001).

Again, there was no main effect of condition on velocity (F = 0.359, p = 0.701, partial ŋ^2^ = 0.018), nor time × condition interaction effect (F = 0.712, p = 0.681, partial ŋ^2^ = 0.034). However, there was a main effect of time (F = 10.802, p ≤ 0.05, partial ŋ^2^ = 0.213), with large effect size. Velocity was significantly higher at 3 min, 6 min and 9 min compared to baseline (p ≤ 0.05).

In contrast, there were no main effects of condition (F = 0.410, p = 0.666, partial ŋ^2^ = 0.020), nor time × condition interaction effect (F = 0.110, p = 0.896, partial ŋ^2^ = 0.005) for average force. However, there was a main effect of time (F = 2.568, p ≤ 0.05, partial ŋ^2^ = 0.060), with medium effect size. Average force was significantly higher at 1 min, 3 min, and 9 min compared to baseline (p ≤ 0.01).

**FIG. 3:**
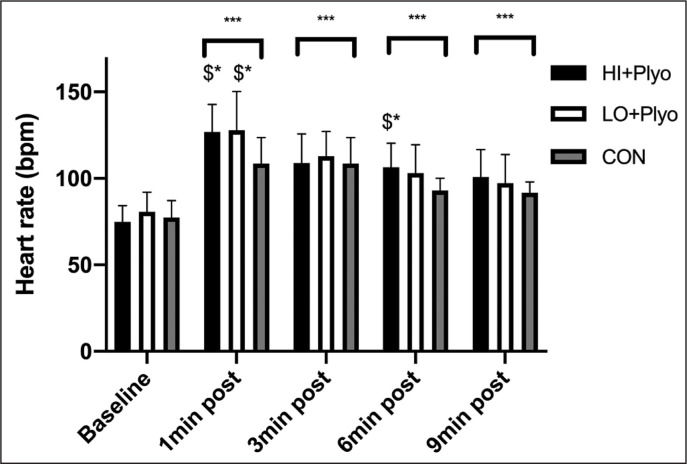
Results for heart rate for each warm-up condition. $ = Different from control condition, bracket = all conditions different from baseline. *p ≤ 0.05; ***p ≤ 0.001.

Finally, there was no main effect of condition on HR (F = 2.648, p = 0.083, partial ŋ^2^ = 0.114). However, there was a significant main effect of time (F = 49.347, p ≤ 0.001, partial ŋ^2^ = 0.839) and time × condition interaction effect (F = 3.044, p = 0.005, partial ŋ^2^ = 0.238), with large effect sizes. As shown in Figure 2, post-hoc comparisons revealed that HRs were significantly higher (p ≤ 0.01) at 1 min, 3 min, 6 min and 9 min compared to baseline. Furthermore, following 1 min of recovery, HRs were significantly (p ≤ 0.05) lower in CON compared to HI + PLYO, LO + PLYO and CON. Following 6 min of recovery HRs were also significantly lower in CON than HI + PLYO (p ≤ 0.05), with no further significant differences between conditions at other time-points (Figure 3).

## DISCUSSION

The main findings of the present study indicate that combined cycling and plyometric activity acutely enhances CMJ height in recreationally active males between 1 min and 9 min post-stimulus, compared to no significant effect of a lower intensity cycling control condition. However, there was no evidence that one experimental condition was superior over the other, suggesting that the plyometric aspect may be the crucial component. The only slight difference between experimental conditions was that HR remained elevated 6 min post-stimulus following the higher intensity cycling intervention. It is noted that there were no differences in how elevated HR (vs. CON) was between HI + Plyo and LO + Plyo 1 min post-stimulus, again suggesting the plyometric aspect may have been predominant in dictating the intensity of the conditioning stimulus.

There is good evidence of a performance-enhancing effect from the preconditioning activities, and this could in part be due to an increase in the peak force and rate of force development of a twitch contraction [28, 29], mainly due to an increase in calcium sensitivity of the acto-myosin complex caused by phosphorylation of the myosin regulatory light chain occurring in type II muscle fibres. PAP is a well-described phenomenon with a short half-life (~28 s) [30], usually < 3 min. However, the time course of myosin regulatory light chain phosphorylation rarely matches that of voluntary force enhancement, and other changes such as muscle temperature, muscle/cellular water content and muscle activation may underpin voluntary force enhancement [23] (called PAPE), so the time-course and mechanisms differentiate PAPE from PAP. In our study, the improvements of CMJ were found in both EXP conditions at 1 min post warm-up, in line with a PAP effect. However, improvements at later time points could be due to a PAPE effect (3–9 min). We consider it unlikely that improved CMJ performance was due to a learning effect as there was no change in the CON condition, and three maximal CMJs were performed before the baseline CMJs with no differences evident and the subjects had previous experiences in this action. It is worth noting that there has previously been mixed evidence regarding PAPE effects in less trained subjects such as ours, so the current findings are meaningful. An important contributing factor may be the specificity of force direction of the conditioning stimulus and muscles and movement involved (vertical plyometrics) to the outcome measure (vertical CMJ), as has been highlighted in more trained participants [13]. For that reason, we consider that the plyometric aspect may be the crucial component due to the specificity of preconditioning activity with the outcome measure (CMJ).

A previous meta-analysis and systematic review about PAP [4] showed that conditioning activity augmented power output, and these effects increased with training experience. In addition, potentiation was optimal following multiple sets at moderate intensities (60–85% 1RM) and using recovery period lengths between 7–10 min. Previous studies have shown a 4 min recovery duration was better than 5 min compared to baseline vertical jump height [31]. However, as we have observed in our study, a consistent optimal time did not seem to depend on the intensity of previous cycling effort, with similar cardiovascular stress between experimental conditions (Figure 2). This further emphasises that the plyometric component is likely the key aspect determining the intensity of the conditioning stimulus, rather than cycling. Plyometrics using DJ exercise with 70–75 cm box heights have shown acute increases in CMJ height between 5 to 15 min post exercise [32, 33] and in less time post exercise (1 to 5 min) using 20–60 cm [33, 34]. It remains to be determined in our participants if a change in box height from the 40 cm used in the current study would impact on optimal recovery duration.

Analysis of the CMJ kinetic and kinematic variables did not further elucidate which factors were contributing most to the CMJ height improvements. Indeed, only a main effect of time was evident, supporting an overall performance enhancement, though not statistically better than CON. It is likely that a number of factors may have contributed to this potential discrepancy, including several experimental limitations. One clear factor may relate to low statistical power, resulting in a Type II error for any effects of condition. This will have been compounded by variability in jump techniques within and between participants. Previous research on CMJs has shown that improvements in CMJ height can be explained by a variety of differences in jumping strategy and technique [35]. Given that our participants are not high-performance athletes performing CMJs as part of daily monitoring for example, although CMJ height data was consistent (e.g., CON trial data), changes in CMJ technique are likely to have occurred jump-to-jump, resulting in variations in force, velocity and power variables. A further contributing factor is likely that of individual differences, as has been reported extensively in PAP literature [23]. For example, in the HI + Plyo condition, 37.5% of the subjects achieved the higher CMJ height at 1 min, 6.25% at 3 min, 31.25% at 6 min and 25% at 9 min post conditioning activity. However, in the case of the LO + Plyo condition, 25% of the subjects achieved the higher CMJ height at 1 min, 37.5% at 3 min, and 18.75% at both 6 min and 9 min post conditioning activity. Therefore, coaches and athletes must evaluate the individual effect of PAP to achieve the best jump performance. As demonstrated here again, there is high variability between subjects in terms of the optimal recovery time. More studies are needed to analyze the causes of these differences, and therefore, the results must be considered at the individual level. Finally, our results can be applied to physically active men, as women were not included in our study due to the convenience sampling employed.

## CONCLUSIONS

In conclusion, this study has shown that up to 15% CMJ performance enhancement can be elicited following high or low-intensity cycling combined with plyometric preconditioning activities in active males, with the intensity and movement specificity of the plyometric component likely the crucial component. Our results suggest that the optimal recovery time within the 1 min-9 min window may be individualized.

### Practical applications

From a practical viewpoint, we recommended that coaches use combined cycling efforts (high and low-intensity) and drop jumps to acutely improve subsequent CMJ performance. Coaches may wish to explore if there is a consistent optimal recovery time on an individual basis.
